# Supplementary material for: Geographical migration and fitness dynamics of Streptococcus pneumoniae
Source: Nature. 2024 Jul 3;631(8020):386–92. doi: 10.1038/s41586-024-07626-3 (PMC11236706; doi:10.1038/s41586-024-07626-3)
Supplement: Supplementary file 1 — This file contains Supplementary Figs. 1–20 and Supplementary Tables 1–13. [file 41586_2024_7626_MOESM1_ESM.pdf]

nature portfolio

https://doi.org/10.1038/s41586-024-07626-3

Supplementary information

Geographical migration and fitness dynamics of *Streptococcus pneumoniae*

In the format provided by the authors and unedited

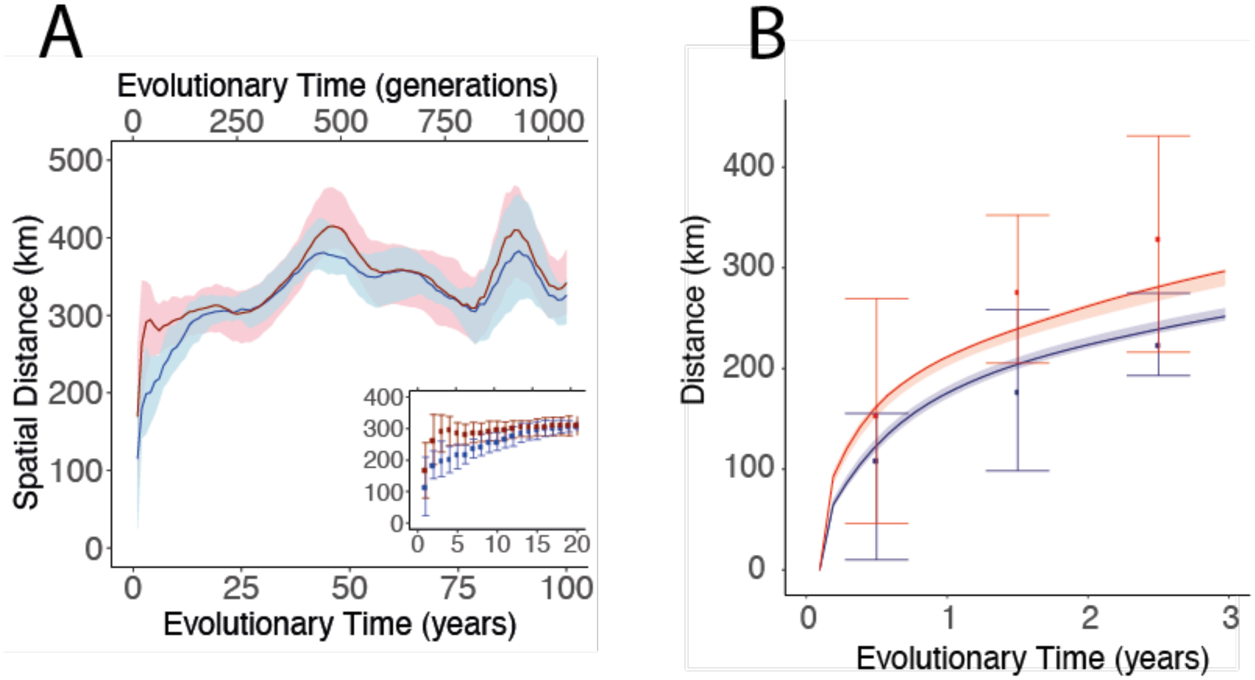

Fig. S1. Geographic distance versus evolutionary time comparing disease alone to carriage and disease. A) Mean geographic distance (km) across rolling 20-year divergence time windows between pairs for disease isolates alone (red) and for all isolates (blue) Inset) Subset to 20 years only. B) Mobility model fit (lines) against data (points) for disease alone (red) and all isolates (blue). Error bars represent 2.5 to 97.5 percentiles resampling the MCMC phylogeny.

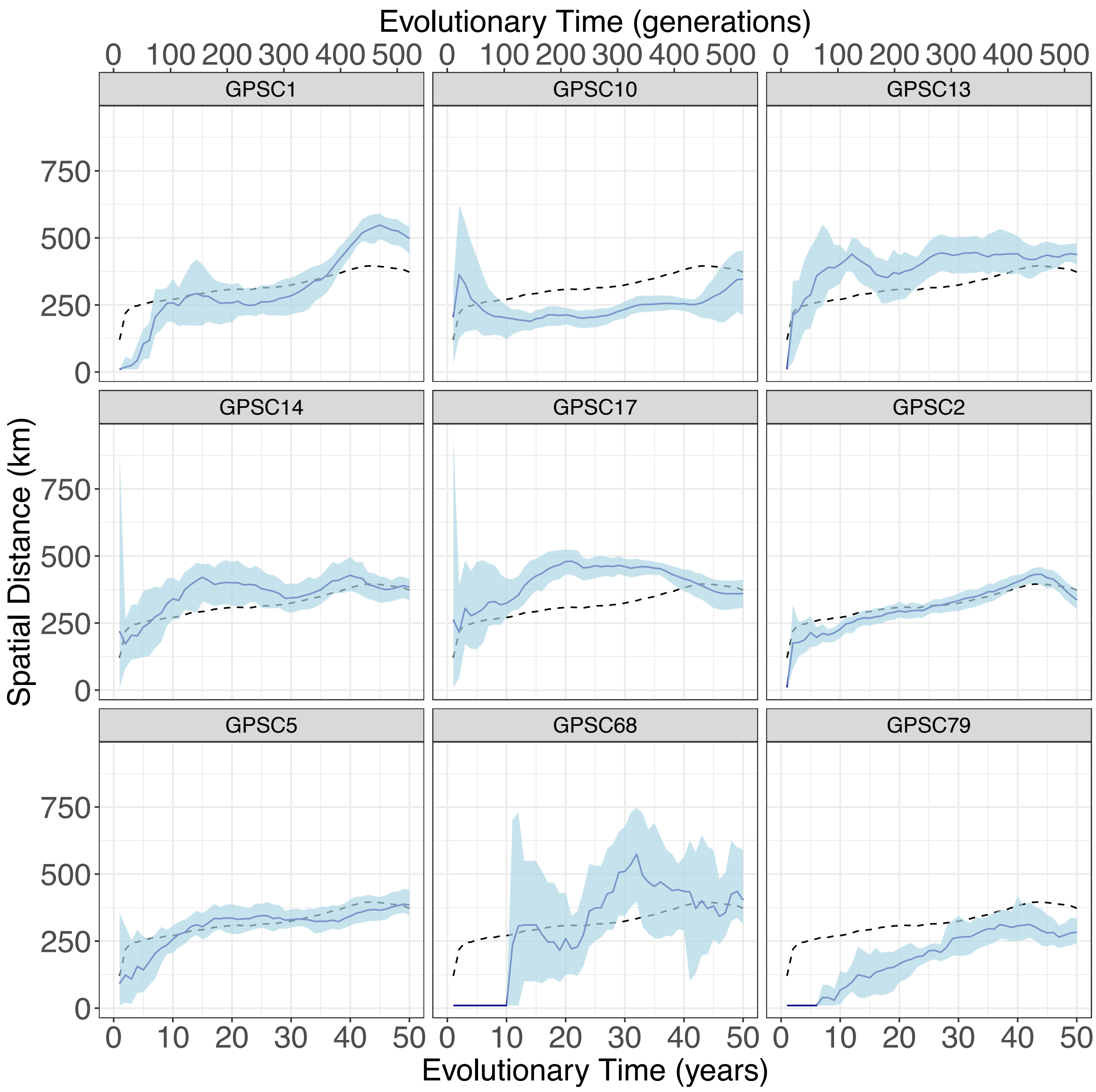

Fig S2. **Geographic distance versus evolutionary time by GPSC in South Africa.** Mean geographic distance (km) across rolling 20-year divergence time windows between pairs for each of the 9 dominant GPSCs (light blue) against the overall line from the aggregated data (purple dashed) using the maximum clade credibility tree. N=2575 (N for each GPSC is listed in Table S4). Error bars represent 2.5 to 97.5 percentiles.

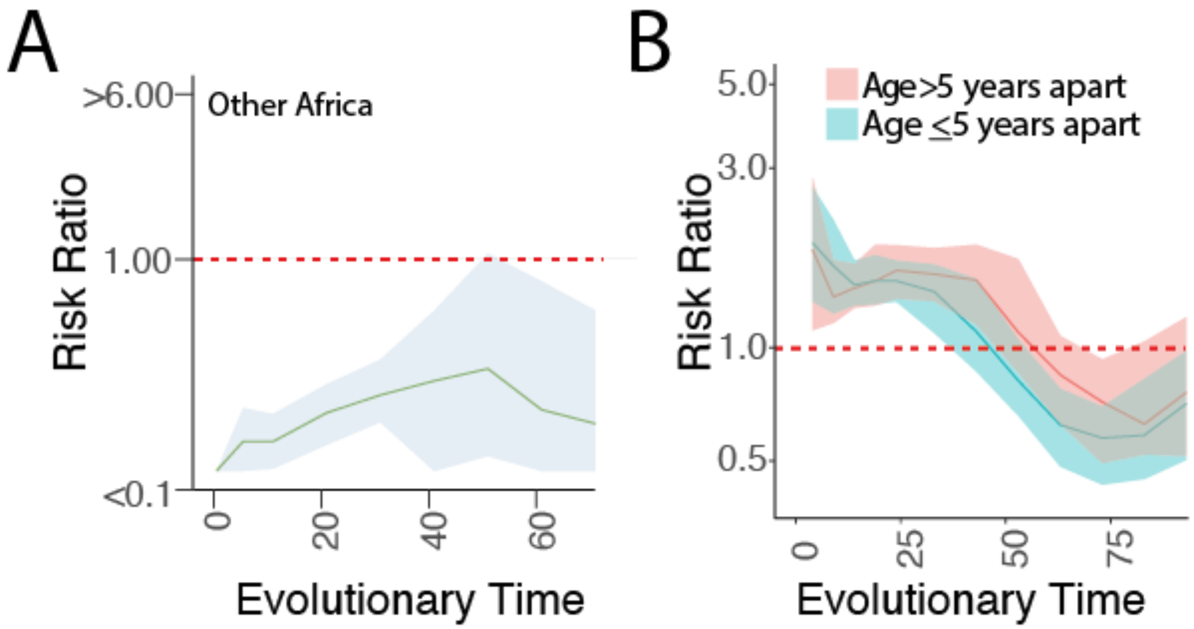

Fig. S3. Relative Risk of similarity over rolling 20-year windows of divergence times for pairs isolated A) for pairs where one isolate is from South Africa and the other is from elsewhere in Africa. B) Relative Risk of similarity over 50-year rolling window of divergence time for pairs isolated from people whose age at collection time was ≤5 years apart (blue) or >5 years apart (pink). Error bars represent 2.5 to 97.5 percentiles across the BactDating posterior.

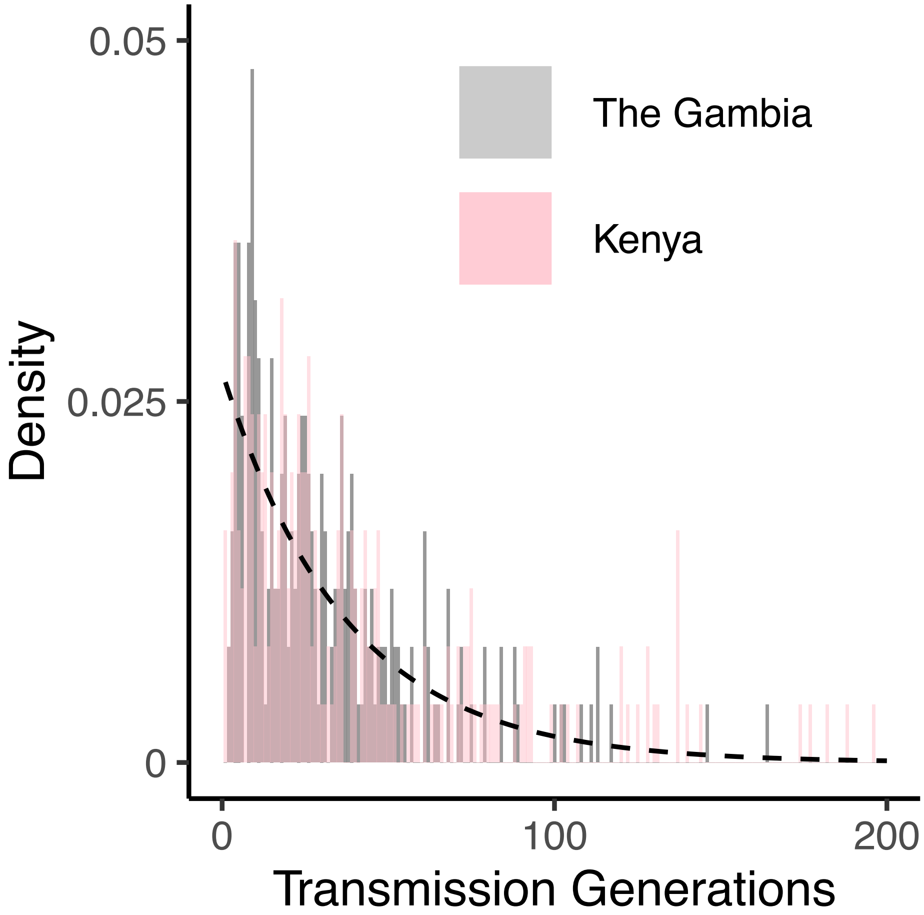

Fig. S4. Transmission generations across gamma distribution. Histogram of simulated transmission generations from The Gambia (grey), and Kenya (pink), overlayed with a gamma distribution curve scaled to the mean of these with a shape of 1 and scale of 0.096 (dashed black).

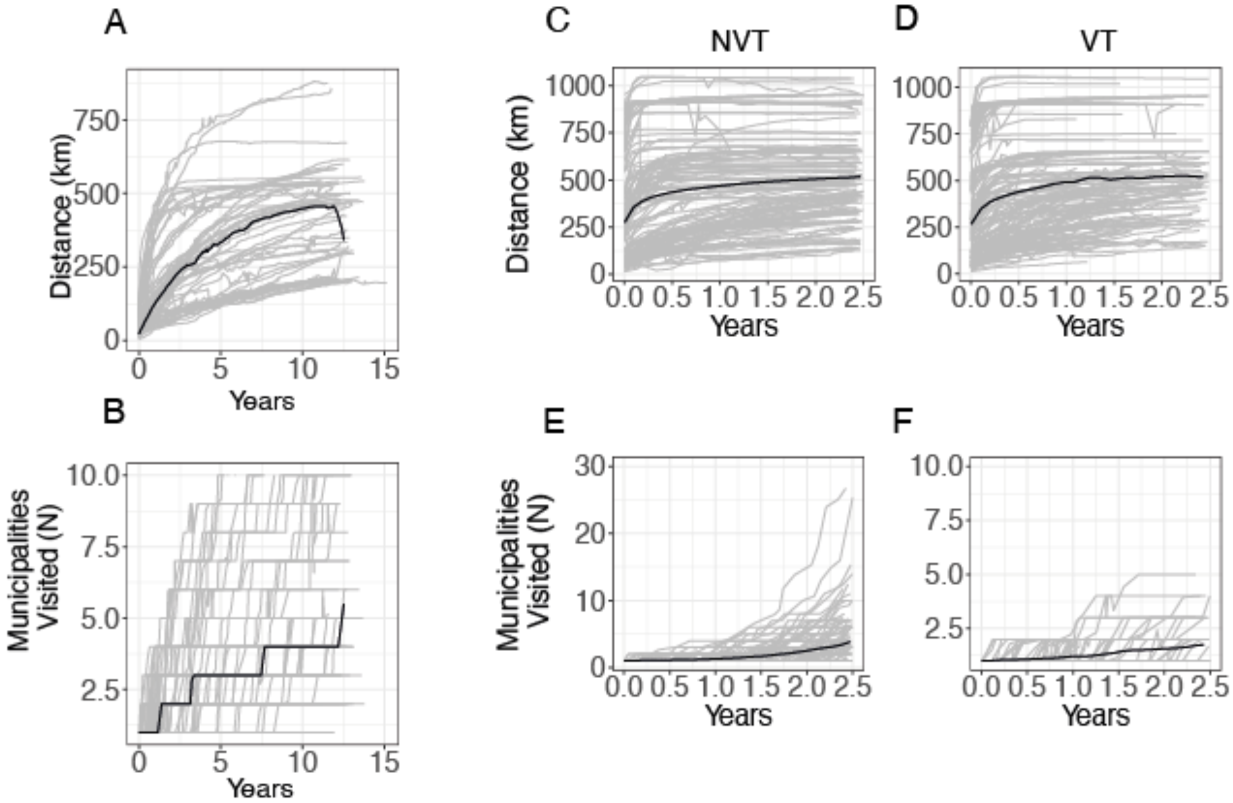

Fig. S5. Branching epidemic simulations adjusting the effective reproductive number (R_{eff})A & B) using a R_{eff} of 1 and a Poisson distribution and C-F) adjusting the R_{eff} by the relative fitness estimated using the fitness model in the post-PCV era. C & E) for NVT serotypes, and D &F) for VT serotypes utilizing the estimated fitness for the metrics A,C,D) distance traveled per simulation, B,E,F) number of unique municipalities visited per simulation. Sampling N=1000.

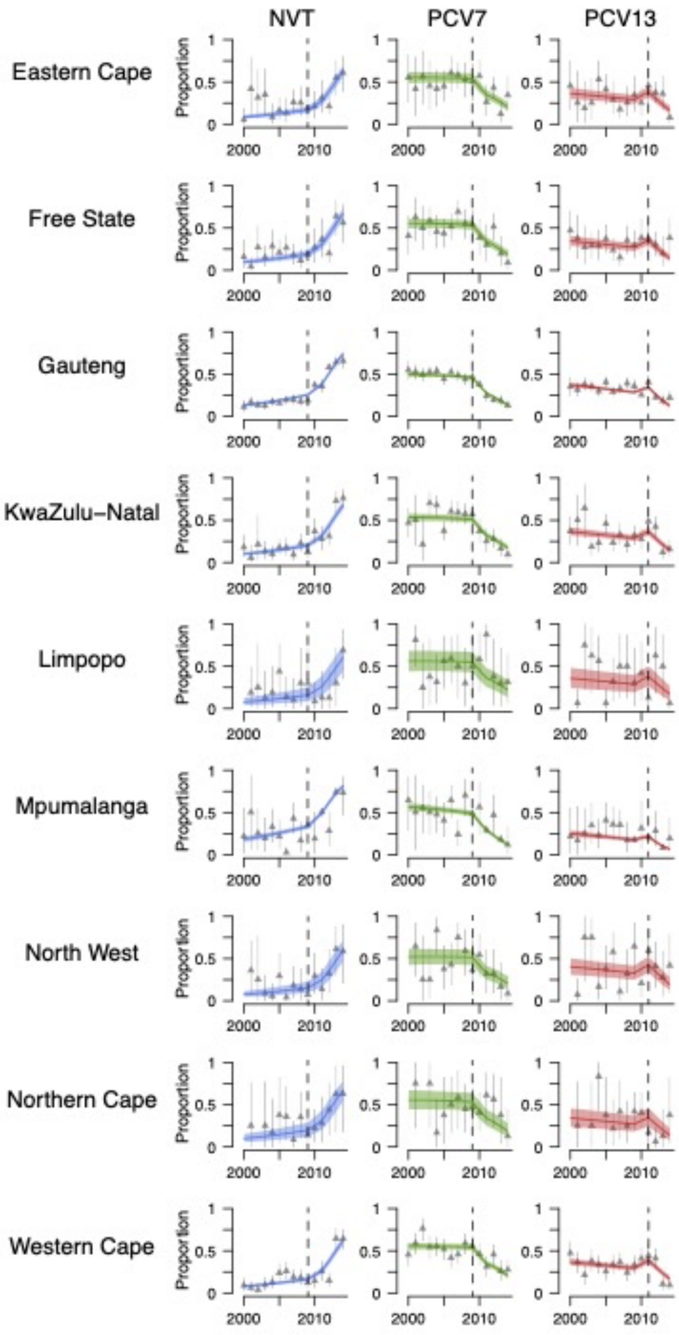

Fig. S6. The proportion of serotype groups across provinces over time from (2000-2014) for NVT serotypes (blue), PCV7 serotypes (green), and PCV13 serotypes (excluding those in PCV7) (red) from the data. The model fits overlay the data including a fitness change in 2009 as indicated by the vertical dashed line. Error bars represent 2.5 to 97.5 percentiles.

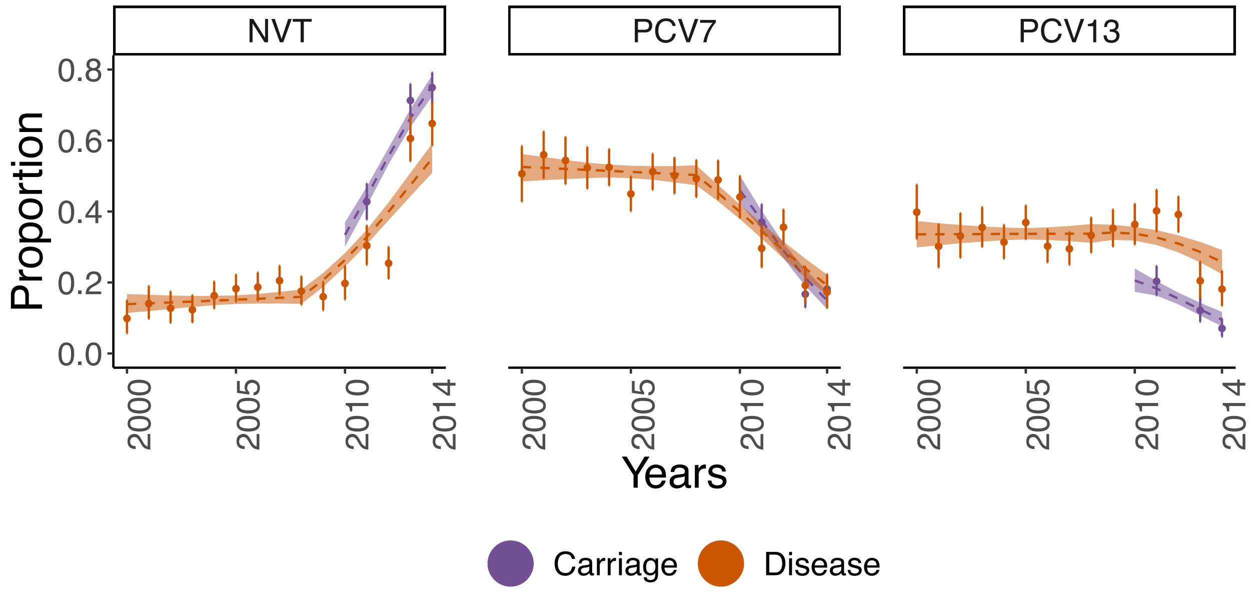

Fig. S7. Comparison of fits for carriage only and disease only isolates. Proportions of groups in the population (points) when looking at only isolates collected from carriage (purple) and isolates collected from disease (orange) overlayed by model fit (lines). Error bars represent 2.5 to 97.5 percentiles.

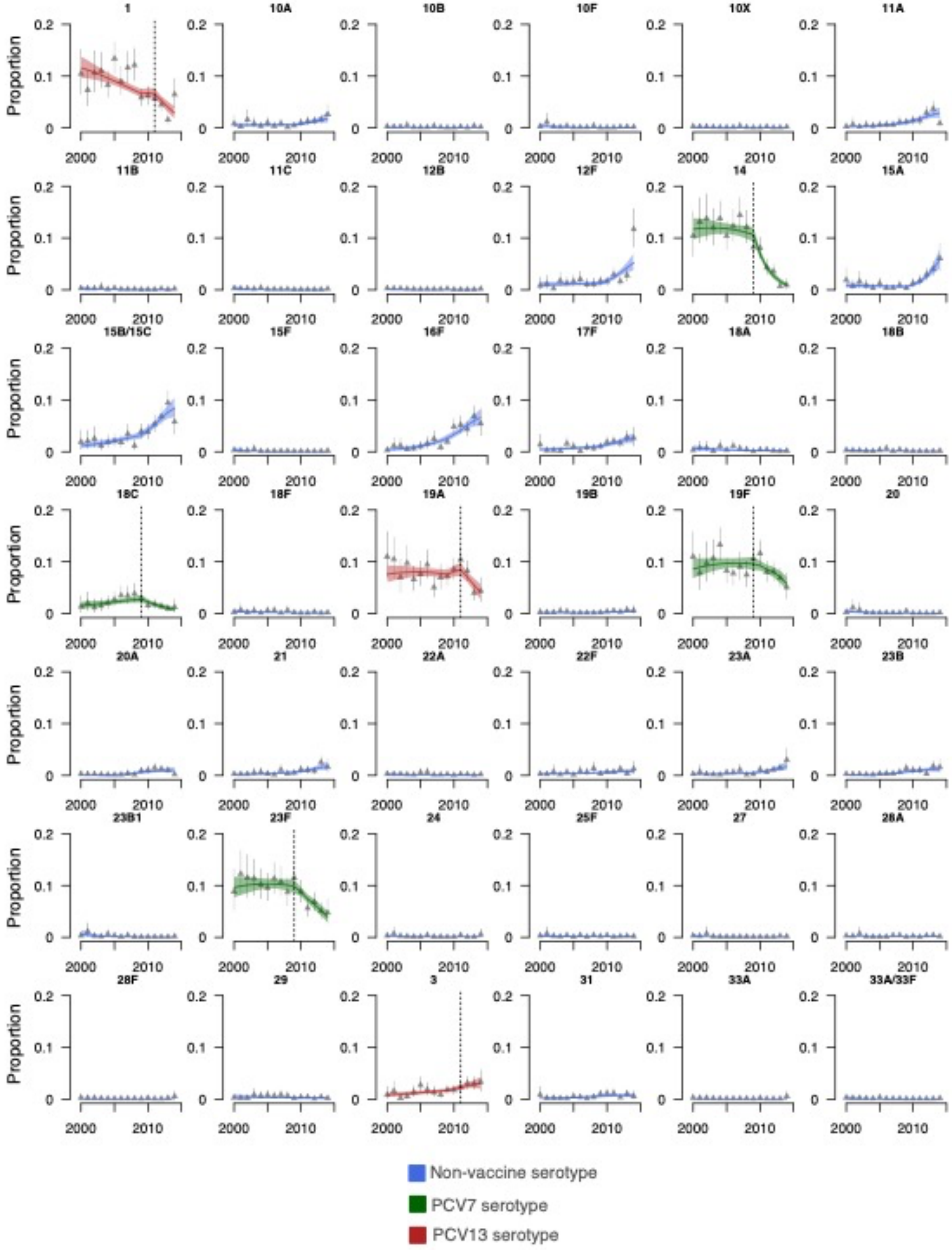
Proportion
1
10A
10B
10F
10X
11A
11B
11C
12B
12F
14
15A
15B/15C
15F
16F
17F
18A
18B
18C
18F
19A
19B
19F
20
20A
21
22A
22F
23A
23B
23B1
23F
24
25F
27
28A
28F
29
3
31
33A
33A/33F
Non-vaccine serotype
PCV7 serotype
PCV13 serotype

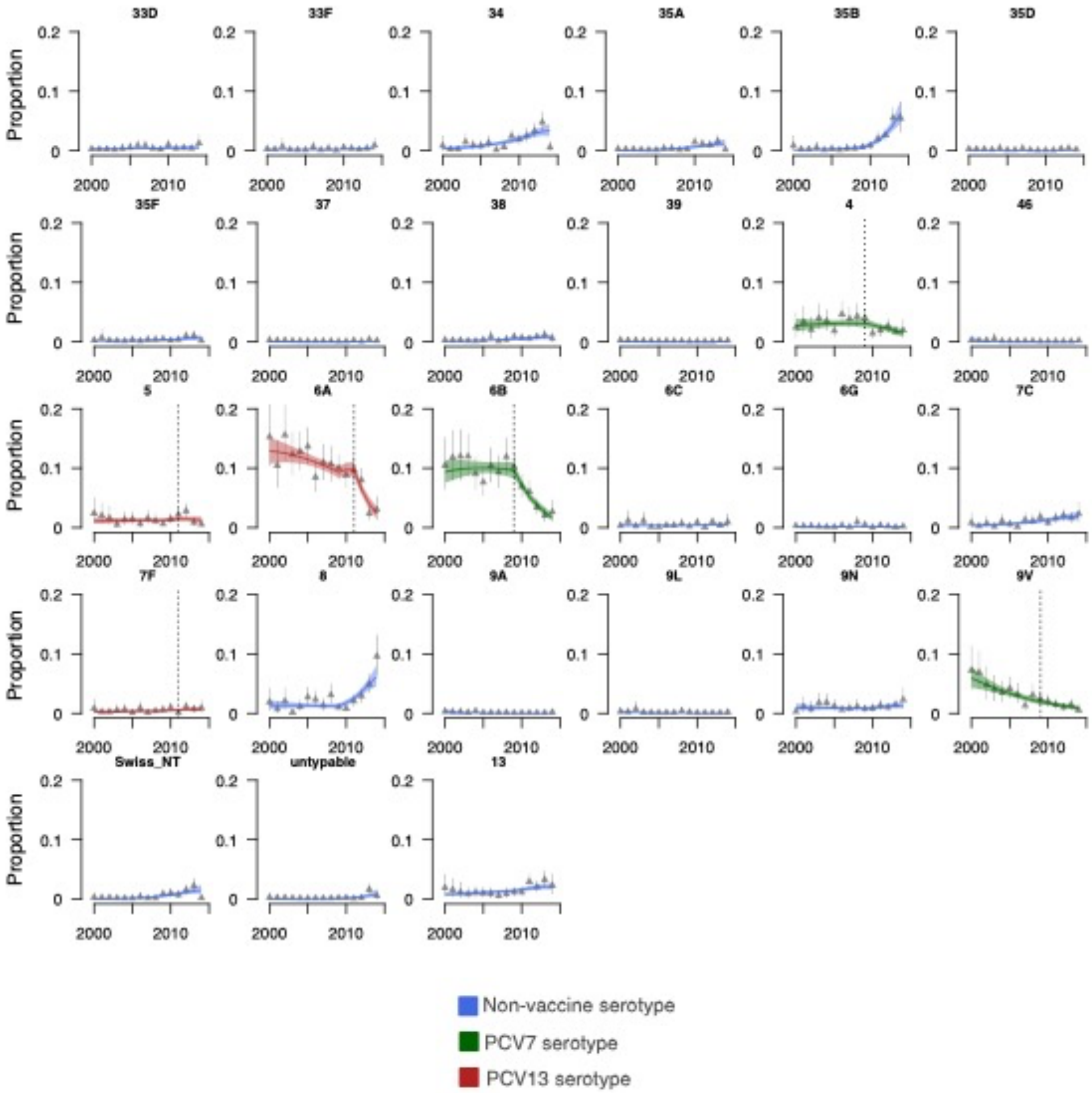

Fig. S8. Serotype fitness estimates. Fits by serotypes colored by whether they are included in PCV7 (green), additionally included in PCV13 (maroon), or not included in any vaccine (blue). Estimating the growth rate prior to PCV7 implementation (2009), and after implementation PCV7 implementation for PCV7 serotypes, and after PCV13 (2011) implementation for PCV13 types. Indicated by the vertical dashed line. Error bars represent 2.5 to 97.5 percentiles.

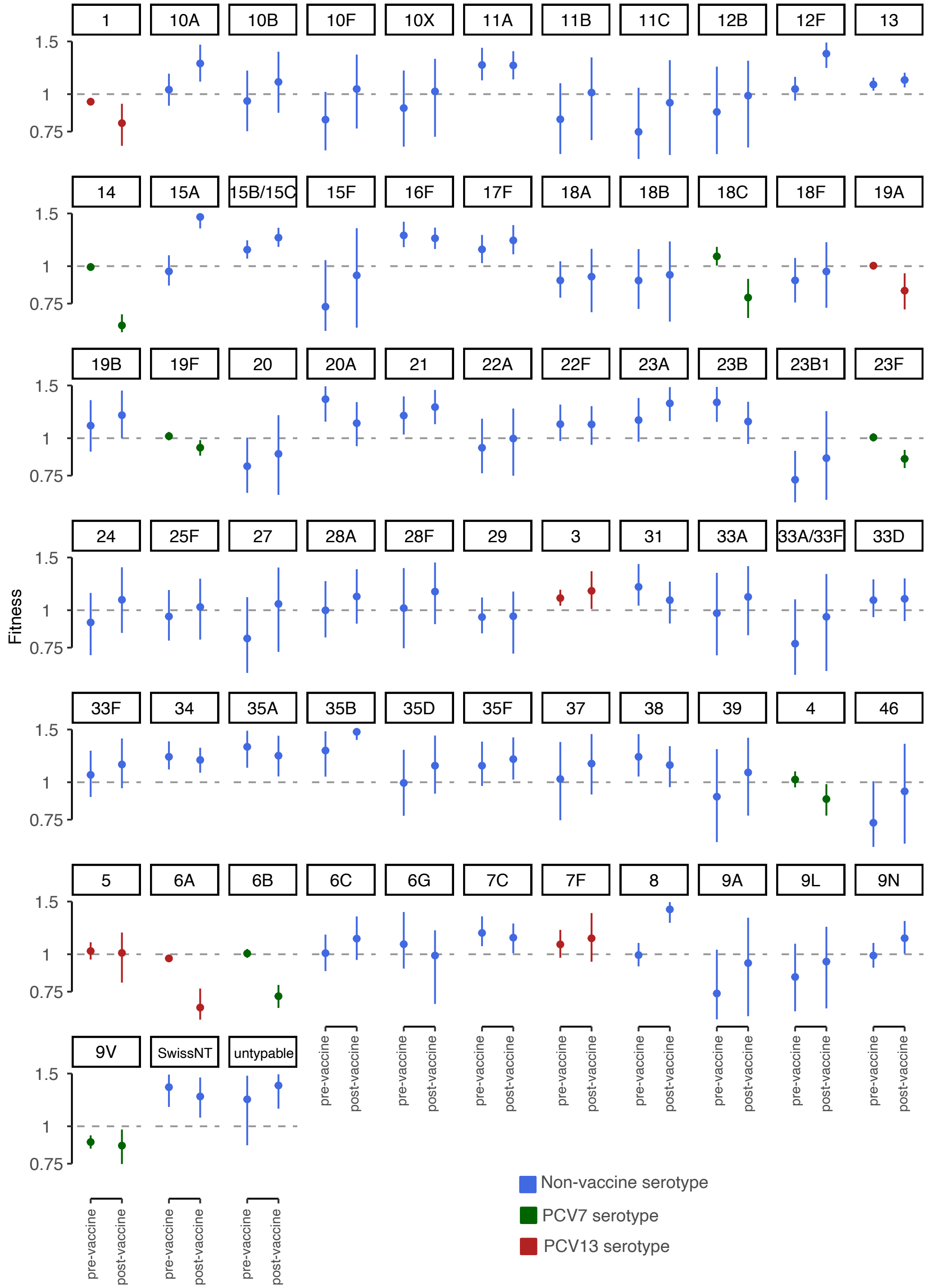

Fig. S9. Fitness estimate for each serotype. Estimates for years prior to PCV7 implementation (2009), and after PCV7 implementation for PCV7 types, and after PCV13 implementation (2011) for PCV13 types. Colored by whether they are included in PCV7 (green), additional types included in PCV13 (maroon), or not included in any vaccine (blue). Error bars represent 2.5 to 97.5 percentiles.

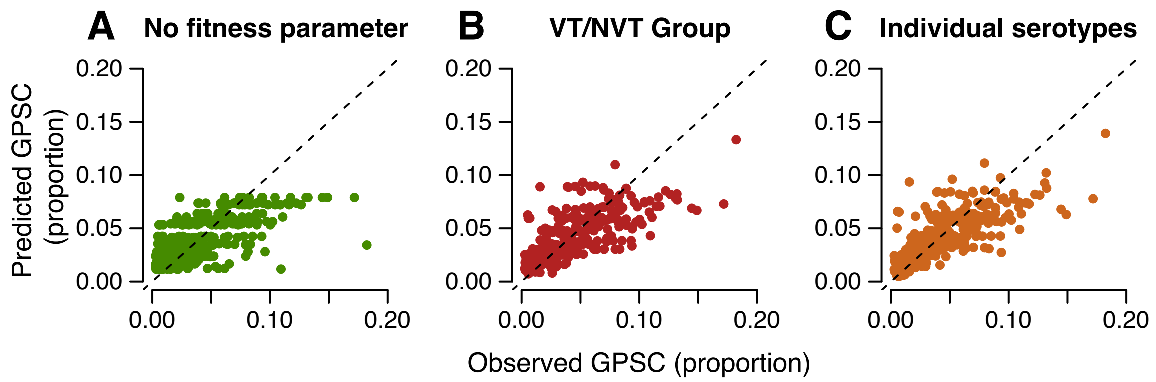

Fig. S10. The observed versus expected R^2 model fits for each GPSC overall, when including A) no fitness parameters (R^2 =0.53)(green) B) VT/NVT fitness parameters (R^2 =0.60) (red) and C) the serotype specific fitness parameters (R^2 =0.65)(orange)

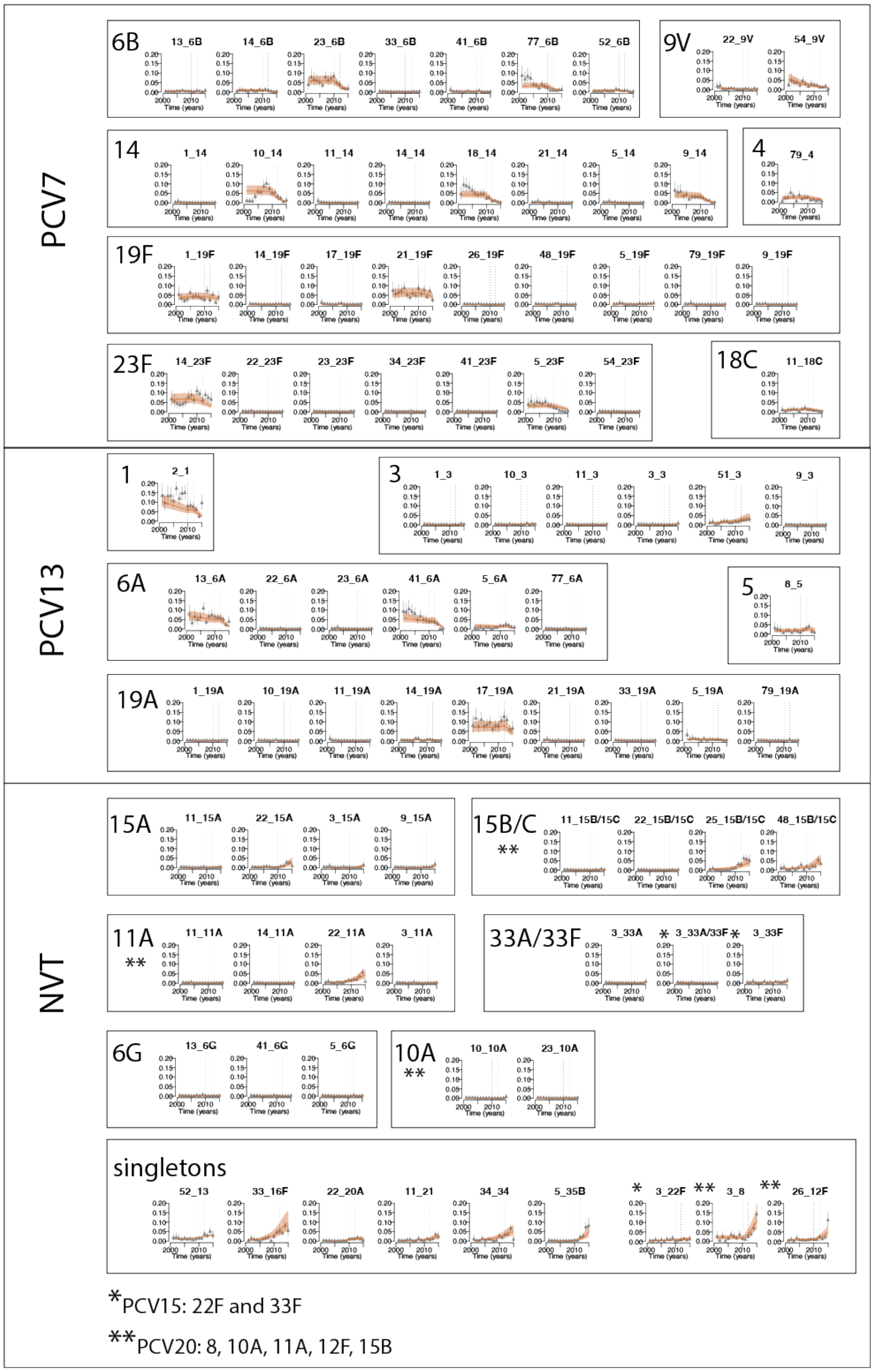

Fig. S11. GPSC-Serotype fits using the serotype specific fitness parameters. The fits are for each GPSC-serotype pair for all pairs where the same serotype appears across multiple GPSCs. These are grouped by PCV7 serotypes, PCV13 serotypes, and NVTs. NVT serotypes with a proportion <0.05 in 2015 which appear in only a single GPSC are excluded. Neither PCV15 or PCV20 are in use in South Africa in 2023.

* serotypes included in PCV15 (Merck)

** indicates serotypes included in PCV20 (Pfizer).

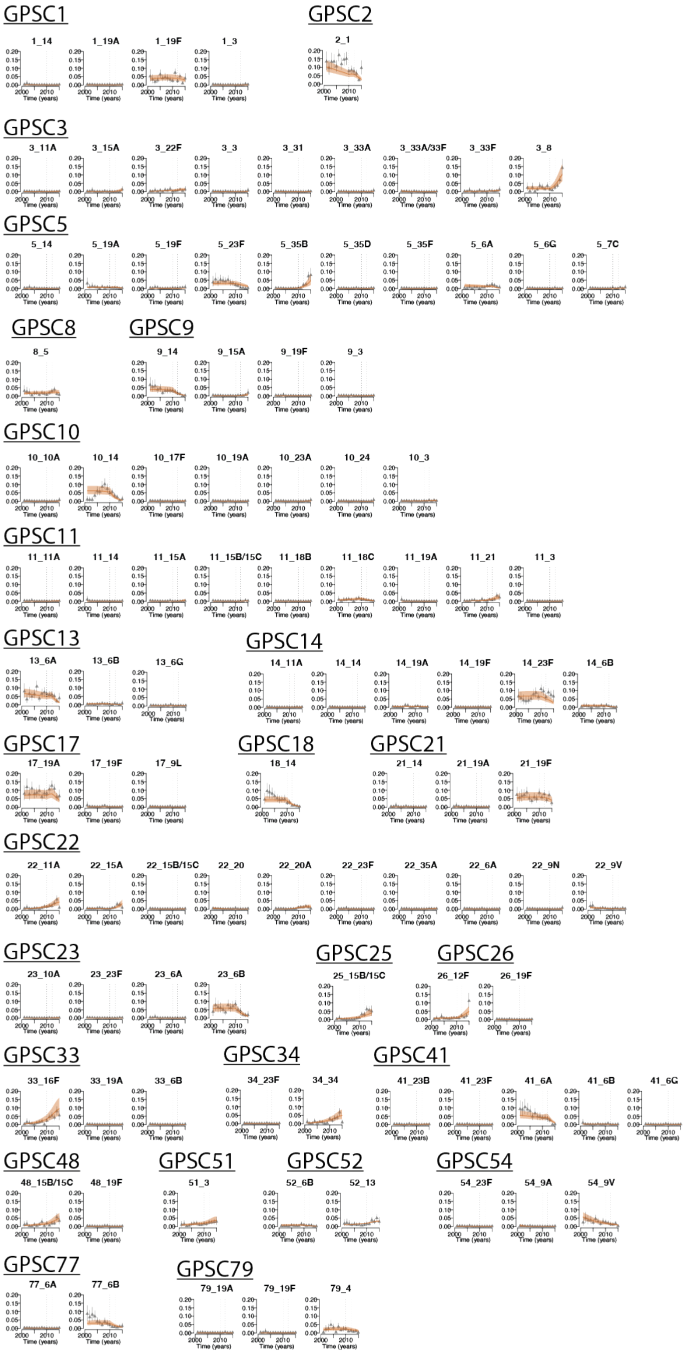

Fig. S12. GPSC-Serotype fits using the serotype specific fitness parameters. The fits are for each GPSC-serotype pair for all pairs where the same serotype appears across multiple GPSCs. These are grouped GPSCs.

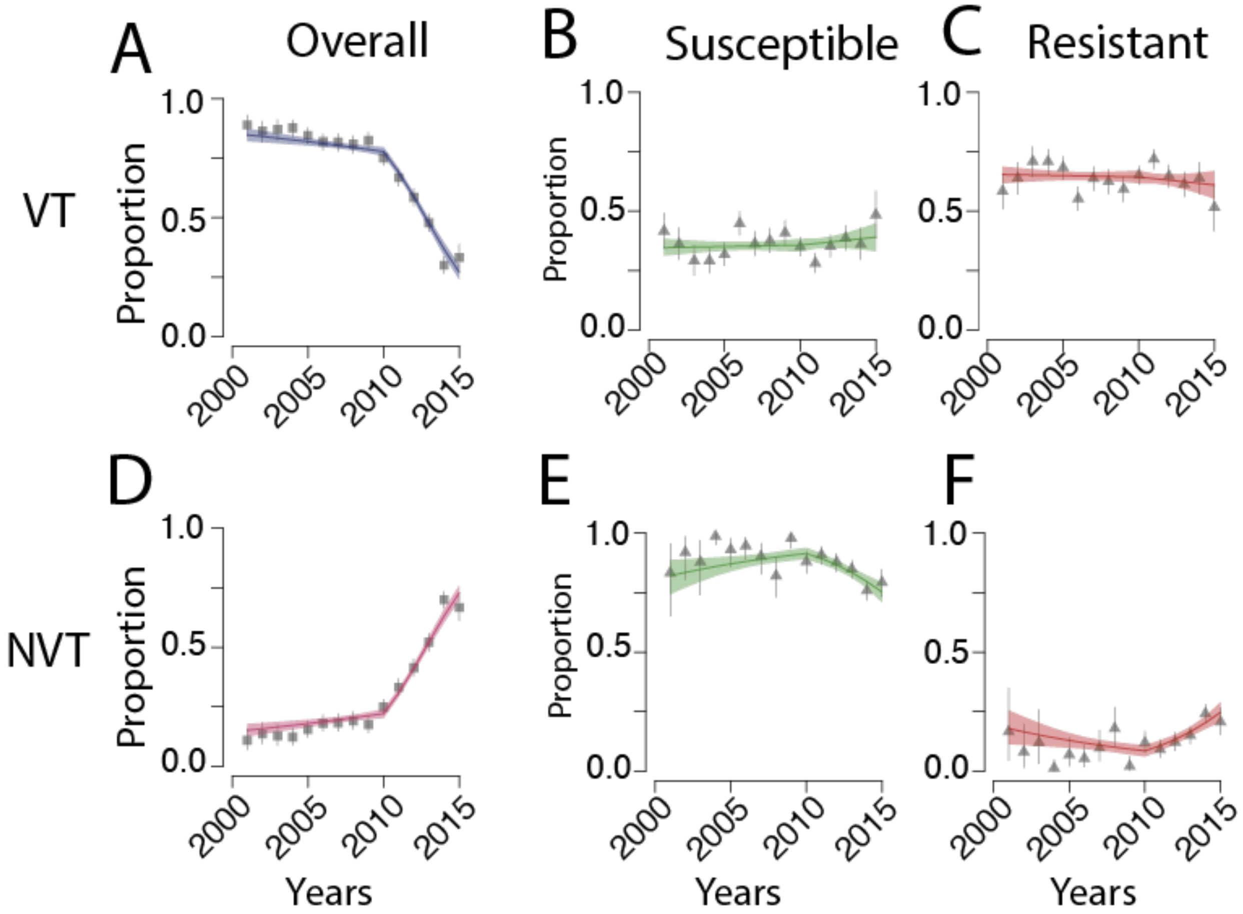

Fig. S13. Fits for the individual proportions over time in the vaccine status & AMR model. Proportion of each group changing over time for VTs (A-C), and NVTs (D-F). A) The proportion change of VT over time included with model fits and D) is the proportion change of NVT over time. Additionally, we include the proportion of (B&E) penicillin susceptible (green) and (C&F) penicillin resistant (red) isolates in each of those groups (NVT and VT) over time with model fits. Error bars represent 2.5 to 97.5 percentiles.

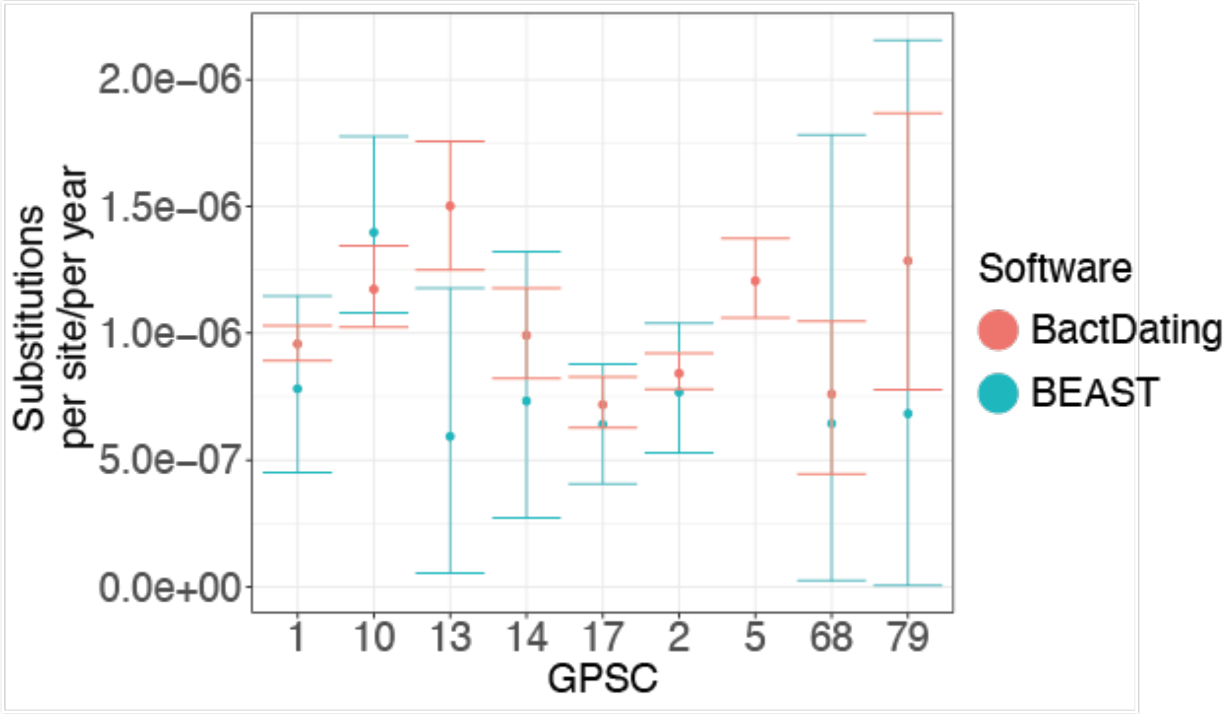

Fig. S14. Parameter comparison between BEAST and BactDating. Comparing the substitutions per site per year from a BactDating relaxed clock model with recombination masked by Gubbins and BEAST relaxed clock model for each of the 9 GPSCs. BEAST was intractable for the diversity in GPSC5. Error bars represent 2.5 to 97.5 percentiles.

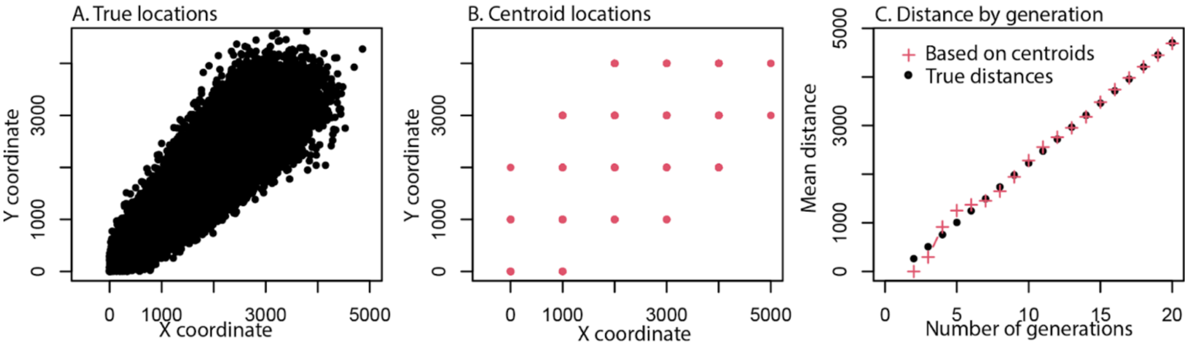

Fig. S15. Comparing centroid distance estimates to true distances. To demonstrate the suitability of using centroid distances, we simulated a spatial transmission process for 1000 separate chains where at each generation a daughter point is placed at a randomly located location 350m in each of the x and y direction. A) This is repeated over 20 generations. B) We then identify the 'centroid' of each case based on the closest coordinate rounded to the nearest kilometre C). We then calculate the total distance covered for both the true distance (black) and the centroid distances (red).

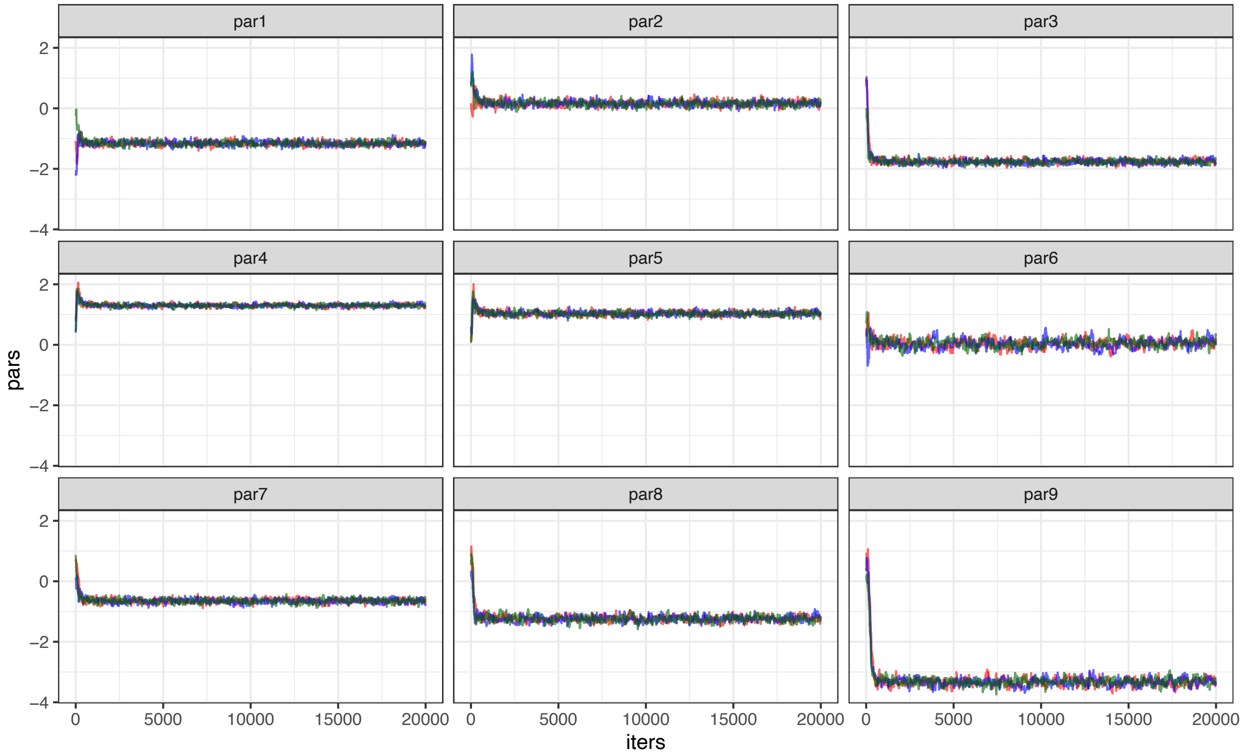

Fig. S16. Chain convergence. Converging chains across 20000 simulations for 9 parameters estimated in the model.

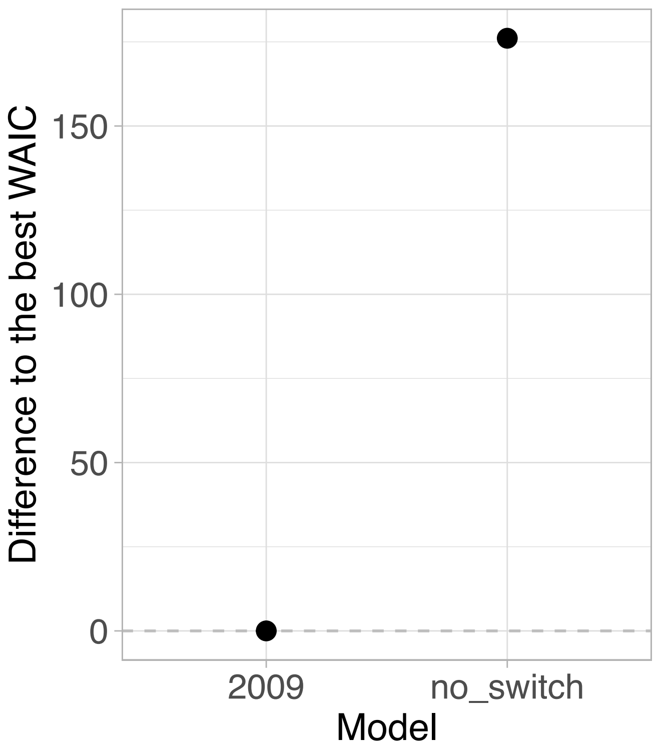

Fig. S17. Model comparison for penicillin susceptibility fitness change model. Estimating fitness for penicillin resistance among VTs and NVTs over time. We include a model with a change in fitness in 2009 upon PCV7 implementation (2009) and a model with no switch (no_switch) in fitness. We present the WAICs.

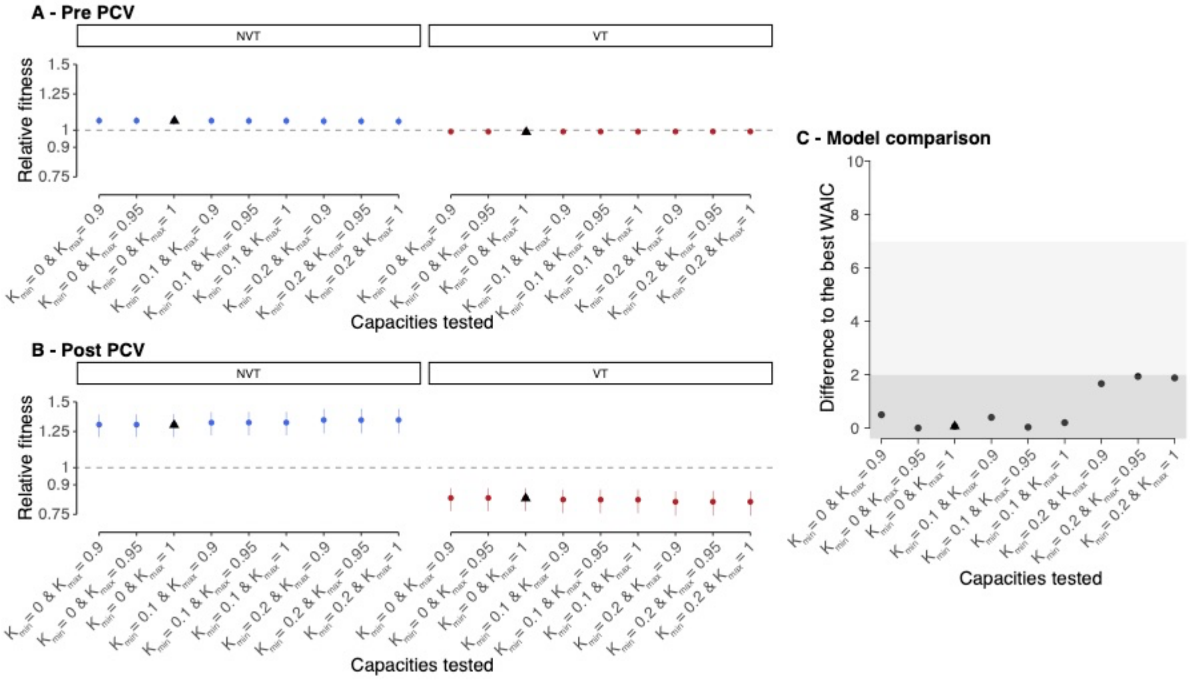

Fig. S18. Fitness estimates with varying carrying capacity across VT and NVT serotypes A) pre-PCV, B) post-PCV introduction, C) and testing the difference to the best WAIC for each. The black triangle represents the carrying capacity utilized in the main model.

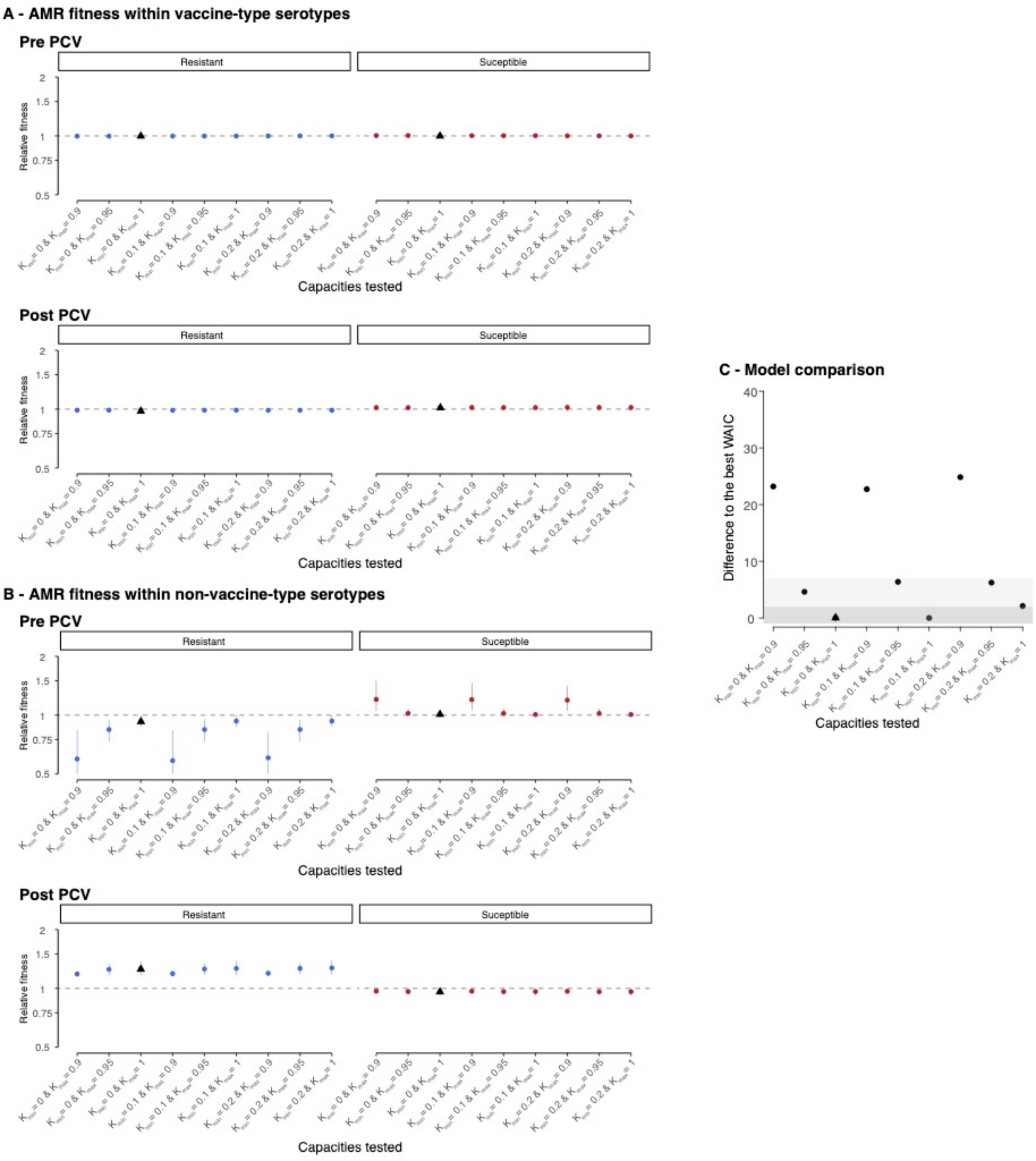

Fig. S19. Fitness estimates with varying carrying capacity for penicillin resistant isolates across A) VT serotypes and B) NVT serotypes where the top row for each is the relative fitness pre-PCV across carrying capacities. and the bottom row is post-PCV introduction relative fitness estimates. C) Testing the difference to the best WAIC for each. The black triangle indicates the carrying capacity utilized in the main model. The dark gray box highlights equivalent models (ΔWAIC $\leq$ 2) and light gray box highlights similar models (ΔWAIC $\leq$ 7). Error bars represent 2.5 and 97.5 percentiles.

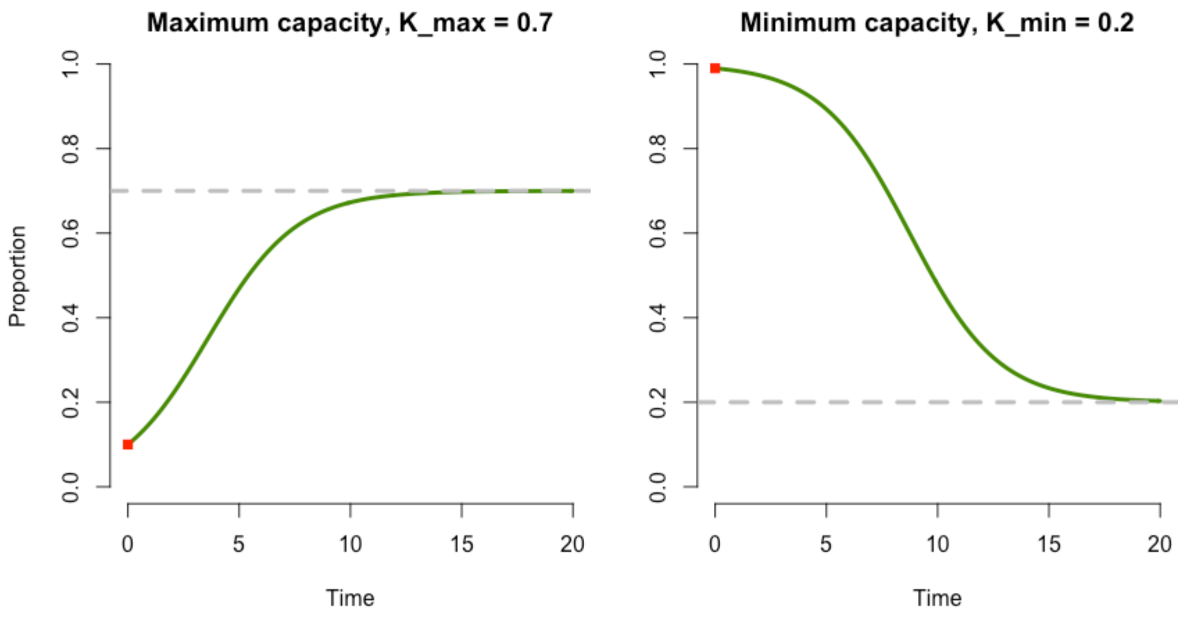

Fig. S20. Example of fitness model dynamics with different carrying capacities.
(left) Maximum capacity of 70%, and initial proportion of 10%. (right) Minimum capacity of 20%, with initial proportion of 99%. Green lines denote the model dynamics. Red squares denote the initial proportions in the population. Dashed lines denote the respective set capacities.

Table S1. South African pneumococcal isolate summary. Summary table of isolates from the 9 provinces of South Africa including the collection years, total number, pre and post-PCV, isolates from patients with invasive pneumococcal disease (IPD), isolates which are not included in the vaccine (NVTs), and dominant GPSCs.

Province	Collection Years	Total	pre-PCV7	post-PCV	IPD		GPSCs	NVT Serotypes		pre-PCV NVT		post-PCV13 NVT		Dominant GPSCs	
		N			N	% IPD	N	N	% Disease	N	% NVT	N	% NVT	N	% Disease
Gauteng	2000-2014	3161	1559	1602	2424	76.7	149	994	31.45	251	16.10	743	46.38	1287	83.6
Western Cape	2000-2014	880	532	348	880	100	85	184	20.91	76	14.29	108	31.03	306	100
KwaZulu-Natal	2000-2014	649	357	292	649	100	77	172	26.5	52	14.57	120	41.10	272	100
Free State	2000-2014	347	219	128	347	100	65	80	23.05	39	17.81	41	32.03	138	100
Eastern Cape	2000-2014	300	149	151	300	100	59	74	24.67	28	18.79	46	30.46	113	100
Mpumalanga	2000-2014	1308	128	1180	194	14.8	111	645	49.31	29	22.66	616	52.20	335	23.6
North West	2001-2014	116	66	50	116	100	40	23	19.8	8	12.12	15	30.00	58	100
Northern Cape	2001-2014	80	27	53	80	100	31	20	25	6	22.22	14	26.42	34	100
Limpopo	2001-2014	69	43	26	69	100	37	14	20.29	7	16.28	7	26.92	32	100
Other Africa	2000-2018	1157	-	-	754	65.2	-	262	22.6	-	-	-			
Other Continents	2000-2015	2944	-	-	1659	56.4	-	481	16.3	-	-	-	-		

Table S2. Isolate breakdown by carriage and disease for the dominant GPSCs used in the phylogenies, the initial dataset used for the lineage level relative risk, dataset included in the fitness analysis and AMR for which in-silico AMR typing was performed.

Year	Dominant GPSCs		Relative Risk Lineage Level		Fitness Analysis & AMR		
	Carriage	Disease	Carriage	Disease	Carriage	Disease	Unknown
2000	0	68	0	186	0	186	0
2001	0	72	0	224	0	224	0
2002	0	75	0	221	0	221	0
2003	0	129	0	313	0	311	2
2004	0	166	0	417	0	417	0
2005	0	215	0	466	0	466	0
2006	0	190	0	419	0	417	2
2007	0	199	0	426	0	425	0
2008	0	186	0	408	0	407	0
2009	102	196	381	381	377	380	0
2010	134	136	365	311	365	310	1
2011	98	136	367	301	360	296	5
2012	73	189	349	425	349	325	7
2013	60	70	388	276	387	267	6
2014	0	81	0	286	0	285	1
Totals	467	2108	1850	5060	1838	4937	23
Overall Totals	2575		6910		6798		

Table S3. Antimicrobial resistance across South Africa for three classes of antimicrobials. The antimicrobials investigated include penicillin (beta-lactam), erythromycin, clindamycin (macrolides), and co-trimoxazole (sulfonamide). Some isolates may be resistant to more than one antibiotic. Antimicrobial-resistance is as predicted by the in-silico antimicrobial resistance pipeline.

Antimicrobial	Resistant (%)	Resistant Carriage (%)	Resistant IPD (%)	Resistant VT (%)	Resistant (N)	NA
Penicillin_WGS	48.18	44.12	49.59	90.35	3273	5
Erythromycin_WGS	17.3	15.63	17.86	93.36	1175	6
Clindamycin_WGS	11.15	7.63	12.41	95.90	757	6
Cotrimoxazole_WGS	68.39	69.76	67.82	81.50	4596	78
Total					6798	

Table S4. GPSCs and their serotypes included in time-resolved phylogenetic trees. Including column 1) lineage, column 2) the total number and column 3) the number from South Africa for each GPSC. Column 4) includes the serotypes each GPSC comprises and column 5) includes their count.

Lineage	Count (overall)	Count (South Africa)	Serotypes (SA)	Serotypes N (SA)
GPSC1	1911	199	19F	193
			19A	3
			14	1
GPSC2	1394	507	1	507
GPSC5	831	300	23F	124
			35B	73
			19A	26
			6A	52
			19F	11
			7C	2
			6G	2
			35D	2
			14	2
			35F	1
GPSC10	709	236	14	223
			3	7
			23A	2
			10A	1
			17F	1
			19A	1
			24	1
GPSC13	598	308	6A	291
			6B	12
			6G	5
GPSC14	512	413	23F	369
			6B	25
			19A	14
			19F	3
			14	1
			11A	1
GPSC17	531	465	19A	460
			9L	1
GPSC68	95	64	18C	60
			18B	3
			17F	1
GPSC79	95	83	4	83

Table S5. Relative Risk of pairs being within each distance, stratified by divergence time (years), compared to distant pairs which are >1000km apart. For the by-lineage comparison the denominator is pairs which are from different lineages. We repeated this analysis including only pairs isolated from disease. The 95% credible intervals sample across the BactDating posterior.

**Distance**	Carriage & Disease			Disease			**Divergence Time (years)**
	**mean**	**2.5 % CI**	**97.5% CI**	**mean**	**2.5 % CI**	**97.5% CI**	
**Within Province**	3.868	3.007	5.098	2.743	1.862	3.650	0 - 5
**<500**	1.580	1.290	2.268	1.723	1.159	2.307	0 - 5
**500-1000**	1.623	1.201	2.365	1.392	0.943	1.917	0 - 5
**Distant Pairs**	1.018	1.002	1.033	1.011	0.997	1.018	0 - 5
**Other Africa**	0.038	0.008	0.095	<0.100	<0.100	<0.100	0 - 5
**Outside Africa**	0.003	0.001	0.012	<0.100	<0.100	<0.100	0 - 5
**Within Province**	3.123	2.344	4.244	2.532	1.958	4.054	5 - 10
**<500**	1.973	1.489	2.703	1.834	1.401	2.605	5 - 10
**500-1000**	1.797	1.327	2.344	1.439	1.041	2.142	5 - 10
**Distant Pairs**	1.014	1.000	1.022	1.002	0.993	1.009	5 - 10
**Other Africa**	0.164	0.074	0.314	0.117	<0.11	0.210	5 - 10
**Outside Africa**	0.015	0.007	0.040	<0.100	<0.100	<0.100	5 - 10
**Within Province**	1.672	1.320	1.997	1.739	1.463	2.136	10 - 20
**<500**	1.361	1.119	1.637	1.507	1.219	1.882	10 - 20
**500-1000**	1.397	1.254	1.727	1.344	1.105	1.577	10 - 20
**Distant Pairs**	1.010	1.006	1.014	1.003	1.000	1.007	10 - 20
**Other Africa**	0.123	0.084	0.195	0.137	<0.100	0.184	10 - 20
**Outside Africa**	0.374	0.116	0.739	0.489	0.199	0.865	10 - 20
**Within Province**	0.918	0.792	1.046	0.886	0.742	1.010	20 - 200
**<500**	1.053	0.870	1.156	1.004	0.812	1.152	20 - 200
**500-1000**	0.959	0.870	1.059	0.959	0.851	1.060	20 - 200
**Distant Pairs**	1.000	0.996	1.003	0.999	0.996	1.000	20 - 200
**Other Africa**	0.689	0.338	1.333	0.427	0.251	1.324	20 - 200
**Outside Africa**	0.702	0.487	0.945	0.525	0.386	0.991	20 - 200
**Within Province**	1.268	1.230	1.308	1.387	1.336	1.430	Same Lineage
**<500**	1.010	0.980	1.042	1.229	1.166	1.286	Same Lineage
**500-1000**	1.085	1.025	1.122	1.139	1.093	1.198	Same Lineage
**Distant Pairs**	1.005	1.004	1.006	1.001	1.001	1.002	Same Lineage

Table S6. Relative Risk of similarity across different distances and for a rolling window of relatedness (tMRCA). The 95% confidence intervals sample across the BactDating posterior.

MRCA		Carriage & Disease			Disease		
**range**	**median**	**RR**	**2.5 % CI**	**97.5% CI**	**RR**	**2.5 % CI**	**97.5% CI**
0 - 1	0.5	3.995	2.186	8.442	2.460	1.587	4.623
0 - 11	5.5	3.225	2.595	4.500	2.665	2.177	3.538
0 - 21	11	1.962	1.744	2.231	1.864	1.664	2.219
0 - 31	21	1.636	1.472	1.888	1.606	1.394	1.921
21 - 41	31	1.703	1.458	2.135	1.712	1.367	1.991
31 - 51	41	1.792	1.110	2.158	1.492	1.006	2.237
41 - 61	51	1.101	0.631	1.996	0.926	0.450	1.969
51 - 71	61	0.546	0.422	1.184	0.525	0.359	0.852
61 - 81	71	0.487	0.350	0.624	0.452	0.335	0.842

Table S7. Age breakdown of isolates. Across All South African isolates (N=6910) and across Dominant GPSCs (N=2575). The percent of isolates in each age breakdown are of those with age data (N=5166 and N=1964 respectively).

Age Group	All South Africa Isolates (N)	All South Africa Isolates (%)	Dominant GPSCs (N)	Dominant GPSCs (%)
≤5	3873	75	1547	78.8
5 - 20	391	7.6	112	5.7
20 - 60	835	16.2	284	14.5
60 - 80	60	1.2	20	1
≥80	7	0.1	1	0.1
Unknown	1744	-	611	-

Table S8. Mobility model comparisons. Model comparisons using Meta human mobility data, distance, and a gravity model with population size (beta) and distance (gamma). This includes the DIC, Pd D_bar, and difference to the best model (DIC_difference)

Model	DIC	pD	D_bar	DIC_difference
Meta Model	12290.97	50.00568	12240.97	0
Gravity Model (gamma)	12424.32	102.12193	12322.19	133.344429
Gravity Model (beta-gamma)	12294.34	94.84881	12199.49	3.368031

Table S9. Transmission risk per municipality. The relative risk of transmission chains being in each municipality after 1 year of transmission for all municipalities with a relative risk >1.

Municipality	Province	RR at 1 year
City of Johannesburg	Gauteng	36.27234
Ethekwini	KwaZulu-Natal	27.36162
City of Cape Town	Western Cape	25.43112
Ekurhuleni	Gauteng	18.83115
City of Tshwane	Gauteng	17.84718
Nelson Mandela Bay	Eastern Cape	6.80979
Buffalo City	Eastern Cape	5.51343
Mangaung	Free State	4.67025
Polokwane	Limpopo	4.57314
Thulamela	Limpopo	3.72567
Mbombela	Mpumalanga	3.54978
Rustenburg	North West	2.92734
Bushbuckridge	Mpumalanga	2.67462
Makhado	Limpopo	2.67033
King Sabata Dalindyebo	Eastern Cape	2.64966
City of Matlosana	North West	2.27253
Emalahleni.mp	Mpumalanga	2.21247
Greater Tzaneen	Limpopo	2.08533
Newcastle	KwaZulu-Natal	2.00226
Greater Tubatse	Limpopo	1.93674
uMhlathuze	KwaZulu-Natal	1.76397
Matjhabeng	Free State	1.75656
The Msunduzi	KwaZulu-Natal	1.70235
Nkomazi	Mpumalanga	1.62864
Mafikeng	North West	1.54947
Govan Mbeki	Mpumalanga	1.27608
Ngquza Hill	Eastern Cape	1.21797
Mogalakwena	Limpopo	1.19574
Emfuleni	Gauteng	1.19145
Nyandeni	Eastern Cape	1.16298
Makhuduthamaga	Limpopo	1.15557
Steve Tshwete	Mpumalanga	1.08615
George	Western Cape	1.06938

Table S10. Absolute fitness estimates. Absolute fitness estimates adjusted by proportion in each group (NVT, PCV7, PCV13 serotypes). Pre-PCV is pre-2009 for NVTs and PCV7 and pre-2011 for PCV13. Error represents the 2.5 and 97.5 percentiles.

	prePCV			postPCV		
Serotype Group	mean	lowerCI	upperCI	mean	lowerCI	upperCI
NVT	1.08	0.16	1.11	1.3	1.18	1.4
PCV7	0.99	0.98	1.01	0.85	0.77	0.92
PCV13	0.97	0.95	0.99	0.74	0.65	0.81

Table S11. Relative fitness estimates by group. Relative fitness estimates as compared to NVT serotypes for both PCV7 and PCV13 (top) and as compared to pre-PCV fitness for each respective group (bottom).

		prePCV			postPCV		
Reference	Serotype Group	mean	lowerCI	upperCI	mean	lowerCI	upperCI
NVT	NVT	1	1	1	1	1	1
	PCV7	0.9	0.88	0.92	0.55	0.49	0.6
	PCV13	0.92	0.89	0.95	0.63	0.6	0.66
Pre-PCV	NVT	1	1	1	1.25	1.14	1.35
	PCV7	1	1	1	0.86	0.78	0.92
	PCV13	1	1	1	0.76	0.67	0.84

Table S12. Absolute fitness estimates by group. Absolute fitness estimates stratified by group for the model incorporating both vaccine status and penicillin resistance profile. This includes pre-PCV and post-PCV. VT is vaccine type serotypes, NVT is non-vaccine type serotypes. R refers to penicillin resistance while S refers to penicillin susceptible.

	pre-PCV			post-PCV			Denominator
Group	mean	2.5% CI	97.5% CI	mean	2.5% CI	97.5% CI	
VT_R	1	1	1	0.99	0.97	1.01	all VT
VT_S	1	1	1	1	1	1.01	all VT
NVT_R	0.99	0.97	1	1.1	1.05	1.15	all NVT
NVT_S	1	1	1.01	0.98	0.96	1	all NVT
VT_overall	0.99	0.98	1	0.89	0.84	0.94	all serotypes
NVT_overall	1.05	1.02	1.07	1.16	1.09	1.24	all serotypes
nvt_s	1.13	1.06	1.22	1.09	1.01	1.18	all serotypes and amr
nvt_r	1.03	1	1.06	1.41	1.33	1.5	all serotypes and amr
vt_s	0.98	0.96	1	0.92	0.85	0.98	all serotypes and amr
vt_r	0.98	0.96	0.99	0.89	0.84	0.94	all serotypes and amr

Table S13. Fitness model summary from three models 1) no fitness parameters (top), 2) the fitness parameters from the VT/NVT model, and 3) the serotype specific fitness parameters (bottom) Model specific information including the number of fitness parameters per model and the AIC are on the left. The middle includes the coefficient of determination for the observed versus expected GPSC-serotype proportions overall and including only the NVT serotypes, the PCV7 serotypes, and the PCV13 serotypes. The final column includes the coefficient of determination for GPSC prevalence overall in the population for each model.

Model Specific Information			GPSC-serotype				GPSCs
Model	Number of fitness parameters	AIC	R^2 - overall	R^2 NVT	R^2 PCV7	R^2 PCV13	R^2
No fitness	100	3730.011	0.74	0.36	0.73	0.82	0.53
VT	106	3351.078	0.78	0.68	0.76	0.81	0.60
Serotype	184	3368.675	0.81	0.77	0.78	0.83	0.65
