# Supplementary material for: Geographical migration and fitness dynamics of Streptococcus pneumoniae
Source: Nature. 2024 Jul 3;631(8020):386–92. doi: 10.1038/s41586-024-07626-3 (PMC11236706; doi:10.1038/s41586-024-07626-3)
Supplement: Supplementary file 2 — Reporting Summary [file 41586_2024_7626_MOESM2_ESM.pdf]

nature portfolio

Corresponding author(s): Sophie Belman

Last updated by author(s): 16/05/2024; May 16, 2024

Reporting Summary

Nature Portfolio wishes to improve the reproducibility of the work that we publish. This form provides structure for consistency and transparency in reporting. For further information on Nature Portfolio policies, see our Editorial Policies and the Editorial Policy Checklist.

Please do not complete any field with "not applicable" or n/a. Refer to the help text for what text to use if an item is not relevant to your study.
For final submission: please carefully check your responses for accuracy; you will not be able to make changes later.

Statistics

For all statistical analyses, confirm that the following items are present in the figure legend, table legend, main text, or Methods section.

n/a	Confirmed	
☐	☒	The exact sample size (*n*) for each experimental group/condition, given as a discrete number and unit of measurement
☐	☒	A statement on whether measurements were taken from distinct samples or whether the same sample was measured repeatedly
☐	☒	The statistical test(s) used AND whether they are one- or two-sided<br>*Only common tests should be described solely by name; describe more complex techniques in the Methods section.*
☒	☐	A description of all covariates tested
☐	☒	A description of any assumptions or corrections, such as tests of normality and adjustment for multiple comparisons
☐	☒	A full description of the statistical parameters including central tendency (e.g. means) or other basic estimates (e.g. regression coefficient) AND variation (e.g. standard deviation) or associated estimates of uncertainty (e.g. confidence intervals)
☐	☒	For null hypothesis testing, the test statistic (e.g. *F*, *t*, *r*) with confidence intervals, effect sizes, degrees of freedom and *P* value noted<br>*Give P values as exact values whenever suitable.*
☐	☒	For Bayesian analysis, information on the choice of priors and Markov chain Monte Carlo settings
☒	☐	For hierarchical and complex designs, identification of the appropriate level for tests and full reporting of outcomes
☐	☒	Estimates of effect sizes (e.g. Cohen's *d*, Pearson's *r*), indicating how they were calculated

Our web collection on statistics for biologists contains articles on many of the points above.

Software and code

Policy information about availability of computer code

Data collection	Population size estimates were collected from LandScan Global 2017 provided by the Oak Ridge National Laboratory (https://landscan.ornl.gov/). Human mobility data was provided by Meta Data for Good, and is available on the Data for Good portals by access request (https://dataforgood.facebook.com/dfg/about).
Data analysis	Computational analysis was conducted in R version 3.6.2 and version 4.0.5 as specified in the GitHub README Bioinformatic analysis was conducted using tools described in the methods section including stan version 2.26.1, VelvetOptimiser v2.2.5, Velvet v1.2.10, SSPACE v2.0, GapFiller v1.11, ABACAS v1.3.1, bwa-MEM v0.7.17, samtools mpileup v1.6, Gubbins v2.4.1 , BactDating v1.0, and BEAST v1.10.4. The MCMC was conducted using fMCMC v0.5-1. All bespoke code is available on GitHub at https://github.com/sophbel/geomig_evo_pneumo

For manuscripts utilizing custom algorithms or software that are central to the research but not yet described in published literature, software must be made available to editors and reviewers. We strongly encourage code deposition in a community repository (e.g. GitHub). See the Nature Portfolio guidelines for submitting code & software for further information.

Data

Policy information about availability of data

All manuscripts must include a data availability statement. This statement should provide the following information, where applicable:

- Accession codes, unique identifiers, or web links for publicly available datasets
- A description of any restrictions on data availability
- For clinical datasets or third party data, please ensure that the statement adheres to our policy

All data are available in the main text or the supplementary materials. All data and code for figures and analysis are accessible at GitHub (https://github.com/sophbel/geomig_evo_pneumo). All whole genome sequences were deposited in the European Nucleotide Database and accession numbers are available in the GitHub repository and on FigShare (doi: 10.6084/m9.figshare.24219214). Associated metadata is available in the Microreact webserver at the following URL: https://microreact.org/project/7wqgd2gbBBEeBLLPKonbaT-belman2024southafricapneumococcus . All scripts for analysis are available on GitHub at: https://github.com/sophbel/geomig_evo_pneumo

Research involving human participants, their data, or biological material

Policy information about studies with human participants or human data. See also policy information about sex, gender (identity/presentation), and sexual orientation and race, ethnicity and racism.

Reporting on sex and gender	N/A
Reporting on race, ethnicity, or other socially relevant groupings	N/A
Population characteristics	N/A
Recruitment	N/A
Ethics oversight	N/A

Note that full information on the approval of the study protocol must also be provided in the manuscript.

Field-specific reporting

Please select the one below that is the best fit for your research. If you are not sure, read the appropriate sections before making your selection.

☒ Life sciences ☐ Behavioural & social sciences ☐ Ecological, evolutionary & environmental sciences

For a reference copy of the document with all sections, see nature.com/documents/nr-reporting-summary-flat.pdf

Life sciences study design

All studies must disclose on these points even when the disclosure is negative.

Sample size	The sample sizes encompass the number of genomes sequenced as part of the Global Pneumococcal Sequencing Project.
Data exclusions	N/A - No data were excluded
Replication	Co-authors and reviewers reviewed the code and verified the reproducibility of the results.
Randomization	N/A - the analysis does not involve experimental groups.
Blinding	N/A - the analysis does not involve experimental groups.

Reporting for specific materials, systems and methods

We require information from authors about some types of materials, experimental systems and methods used in many studies. Here, indicate whether each material, system or method listed is relevant to your study. If you are not sure if a list item applies to your research, read the appropriate section before selecting a response.

Materials & experimental systems

n/a	Involved in the study
☒	☐ Antibodies
☒	☐ Eukaryotic cell lines
☒	☐ Palaeontology and archaeology
☒	☐ Animals and other organisms
☒	☐ Clinical data
☒	☐ Dual use research of concern
☒	☐ Plants

Methods

n/a	Involved in the study
☒	☐ ChIP-seq
☒	☐ Flow cytometry
☒	☐ MRI-based neuroimaging

This checklist template is licensed under a Creative Commons Attribution 4.0 International License, which permits use, sharing, adaptation, distribution and reproduction in any medium or format, as long as you give appropriate credit to the original author(s) and the source, provide a link to the Creative Commons license, and indicate if changes were made. The images or other third party material in this article are included in the article's Creative Commons license, unless indicated otherwise in a credit line to the material. If material is not included in the article's Creative Commons license and your intended use is not permitted by statutory regulation or exceeds the permitted use, you will need to obtain permission directly from the copyright holder. To view a copy of this license, visit http://creativecommons.org/licenses/by/4.0/
